# Duplication Cyst in the Third Part of the Duodenum Presenting with Gastric Outlet Obstruction and Severe Weight Loss

**DOI:** 10.1155/2015/749085

**Published:** 2015-11-15

**Authors:** Osama Shaheen, Samer Sara, Mhd Firas Safadi, Bayan Alsaid

**Affiliations:** ^1^Department of Surgery, Almouwasat University Hospital, Damascus, Syria; ^2^Laboratory of Anatomy, Faculty of Medicine, University of Damascus, Damascus, Syria

## Abstract

Duodenal duplication is a rare developmental abnormality which is usually diagnosed in infancy and childhood, but less frequently in adulthood. We report a case of a 16-year-old female with a duplication cyst in the third part of the duodenum. The patient presented with symptoms of gastric outlet obstruction, including severe anorexia and weight loss. The diagnosis was made preoperatively by CT scan and upper endoscopy. The cyst was successfully treated by marsupialization on the duodenum using a GIA stapler. Duodenal duplication presents with a wide variety of symptoms. Although illusive, many cases can be properly diagnosed preoperatively by using the appropriate imaging modalities. Treatment choices are tailored according to the size and location of the cyst, in addition to its relation to adjacent structures. The outcomes are favorable in the majority of patients.

## 1. Introduction

Duplications of the alimentary tract are rare developmental abnormalities. The overall incidence of this condition is 1 : 25,000, and duodenal duplication constitutes 5–12% of all cases [1]. Most cases of duodenal duplication are seen at the medial border of the first and second portions of the duodenum. Duplication of the third portion is even less frequent [2, 3].

Here, we report a rare case of a duplication cyst that compresses the third part of the duodenum. The cyst presented with gastric outlet obstruction and severe anorexia. To our knowledge, this is the first reported case that presents with severe weight loss and malnutrition in adolescence.

## 2. Case Presentation

A 16-year-old female presented to the emergency department of our hospital complaining of recurrent abdominal pain in the epigastrium especially after meals, with occasional radiation to the left shoulder. She reported a recent history of persistent nausea and vomiting in addition to severe anorexia and weight loss.

The patient looked anxious, dehydrated, and debilitated. She had tachycardia and her BMI was only 15.8 kg/m^2^. Abdominal examination revealed mild tenderness in the epigastrium without rigidity or palpable masses. Laboratory findings showed anemia (hemoglobin 8.2 g/dL), hypokalemia (potassium 3.1 mEq/L), and hypoalbuminemia (albumin 2.2 g/dL). Gastric aspiration yielded about one liter of nonbloody aspirate.

**Figure 1:**
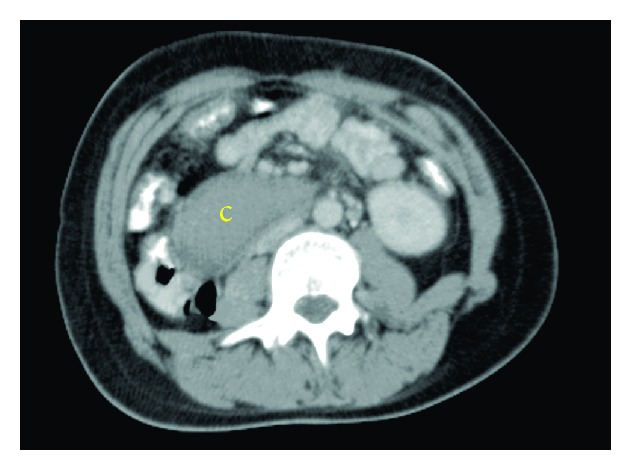
Oral and intravenous contrast-enhanced CT scan of the abdomen. The noncommunicating cyst extends along the third portion of the duodenum (c: cyst).

Abdominal ultrasonography (US) showed severe gastric distension with a big cystic mass in the upper abdomen. Abdominal computed tomography (CT) showed a cystic lesion measuring 8 × 4 × 7 cm, which extends along the third part of the duodenum in the retroperitoneum. The lumen of the third part of the duodenum was completely obscured. The cyst was suspicious for duodenal duplication (Figure 1). Upper endoscopy showed a copious amount of fluids in the stomach with severe gastric dilatation. The third part of the duodenum was nearly occluded, supposedly due to external compression.

**Figure 2:**
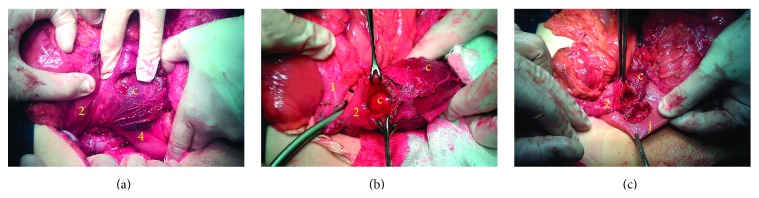
Operative views of the cyst illustrating management steps. (a) Extended Kocher maneuver revealed a big cyst compressing the third part of the duodenum, with close relation to the head of the pancreas. (b) The duodenum was opened through the lateral wall of the second part, which showed the internal common wall between the cyst and the third part of the duodenum. The lumen of the of the third part of the duodenum was completely occluded. The common wall was opened using a GIA-stapler (not shown). (c) The cyst collapsed after marsupialization and duodenojejunal anastomosis was performed (c: cyst, j: jejunum, numbers: corresponding parts of the duodenum).

Upon laparotomy, a cystic structure was found behind the transverse mesocolon. An extended Kocher maneuver was performed, and exploration revealed a big cyst along the third part of the duodenum with close relation to the head of the pancreas (Figure 2(a)). The duodenum was opened on the lower lateral wall, and duodenotomy revealed the cyst which was protruding inside the lumen of the third duodenal portion (Figure 2(b)).

The cyst was opened widely through the common wall, and blood clots were extracted from the cyst. No abnormal pathology was noted inside the cyst. Part of the common wall was resected, and the cyst was marsupialized on the duodenum with the aid of a GIA stapler. Finally, a side-to-side anastomosis was performed between the duodenotomy site and a jejunal loop about 30 cm after the ligament of Treitz (Figure 2(c)).

**Figure 3:**
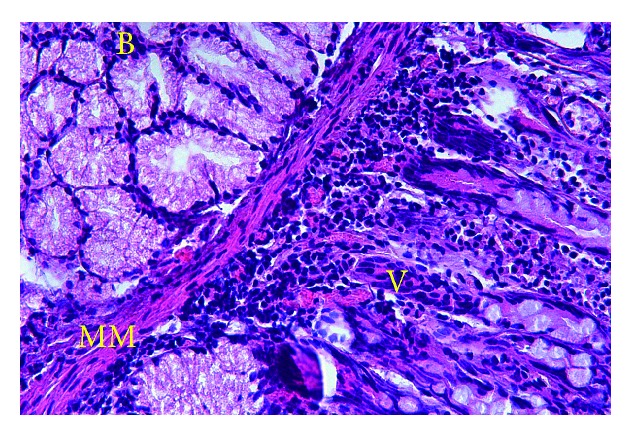
Histological view of the cyst wall showing typical structure of duodenal mucosa (hematoxylin and eosin, ×100) (B: Brunner's glands, MM: mucosa muscularis, V: duodenal villi).

Histopathologic examination confirmed the diagnosis by identifying a duodenal mucosa that lines a smooth muscle coat within the wall of the cyst (Figure 3). The patient was well and free from symptoms after 12 months of follow-up. She regained her nutritional state and her BMI was 20.5 kg/m^2^.

## 3. Discussion

Duplication cysts of gastrointestinal tract were first documented in the mid-19th century by Reginald Fitz [4]. Three criteria are required for the diagnosis of a duplication cyst: an intimate relation to the gastrointestinal tract, the presence of a smooth muscular coat, and the presence of an alimentary mucosal lining [4].

Duodenal duplications represent a low percentage of all duplications. They can be cystic or tubular, and they may communicate with the duodenal lumen, the pancreatic duct, or rarely the biliary system [4]. These cysts often contain mucosal secretions from the epithelial lining, but many authors reported the presence of purulent content or bile [5, 6].

The abnormality is usually diagnosed in infancy and childhood [1]. However, many patients can remain asymptomatic until adulthood, and about one third of patients present after 20 years of age [7].

The clinical features of duodenal duplication cysts vary from asymptomatic cases to nonspecific symptoms such as abdominal pain, abdominal distention, and vomiting [5, 7]. Some patients develop symptoms of gastric outlet obstruction or small bowel obstruction. Ulceration or perforation due to the presence of an ectopic mucosa may cause duodenal bleeding or peritonitis, respectively [8]. Duodenal duplication cysts may also cause recurrent episodes of acute pancreatitis because of the direct pressure applied against the pancreatic duct. Stone formation was reported in some cases due to stasis inside the cyst. Jaundice and intussusceptions have also been reported [6]. Hemorrhagic ascites is a very rare complication of duplication cysts [9].

Most cysts are 2–5 cm in size. The largest diameter of the cyst in our case was 8 cm, which is relatively large. Only few cases in the literature were larger than 5 cm [6]. This was responsible for the severe compressive symptoms which included anorexia, vomiting, malnutrition, and weight loss. The BMI of our patient was 15.8 kg/m^2^ with severe laboratory derangement, and this was not previously reported in any case of duodenal cysts.

The preoperative diagnosis of intestinal duplications is rarely accurate. The differential diagnosis encompasses all cystic lesions in this region, which include choledochoceles, pancreatic pseudocysts, cystic tumors of the pancreas, mesenteric cysts, and duodenal diverticulums [6].

Endoscopic US could differentiate the duplication cyst by the “gut signature” or “the double-layer wall” sign of its wall. CT is valuable in identifying the type, location, and the size of the duplication cyst, and the technetium scan can aid in the detection of heterotopic gastric mucosa in cases complicated with bleeding [10]. Magnetic resonance imaging (MRI) and gastroduodenoscopy are other modalities that can be used for diagnosis. Magnetic resonance cholangiopancreatography (MRCP) is a valuable noninvasive tool for the identification of cysts that communicate with the biliary tree. Endoscopic retrograde cholangiopancreatography (ERCP) was also recommended to visualize the pancreaticobiliary tract and to determine its relationship with the cyst, but ERCP-related complications limit its role [9, 11].

The appropriate surgical procedure for a duodenal duplication cyst depends on its type and location. Consequently, several operative choices are feasible [12]. Total excision is indicated when the cyst is small and not related to the pancreaticobiliary tree or the head of the pancreas, or when the cyst is complicated with an ulcer, either in the cystic mucosa or in the adjacent mucosa. Excision is considered the procedure of choice to avoid possible malignant transformation, which was reported in three cases in the literature [7, 13]. Pancreaticoduodenectomy is rarely required, but it may be the only choice in complicated cysts which are intimately related to the head of the pancreas or the pancreaticobiliary tree [11].

Partial resection of the common wall with internal marsupialization on the duodenum is a good management option to avoid injury to the pancreas, pancreatic and bile ducts, or any related structure as we did in our case. Other possibilities include cystojejunostomy by a jejunal loop or Roux-en-Y anastomosis [8].

The surgeon should judge the best treatment that relieves the symptoms without causing serious complications. Total excision was not possible in our case due to the close relation of the cyst to the head of the pancreas, and marsupialization was a safer option for draining the cyst and relieving the compressive symptoms.

Advances in therapeutic endoscopy, such as endoscopic mucosal resection, were recently used in the management of some duodenal cysts [5]. More recently, many cases of duodenal duplication were managed laparoscopically, including resection or anastomosis, with favorable outcomes [14].

Although duodenal cysts are considered a rare entity, we believe that further research is needed to design a specific classification system and management criteria for these lesions. Such diagnostic and therapeutic criteria would spare many patients the sequelae of laparotomy by taking advantage of the recent advances in minimally invasive and endoscopic techniques. This would only be possible through accumulation of more cases. Therefore, we encourage surgeons to report any lesion of this type with enough details about location and appropriate treatment options.

## 4. Conclusion

Duodenal duplication should be considered in the differential diagnosis of vague upper abdominal symptoms, especially when a cystic structure neighboring the duodenum is demonstrated on radiology. Ideal treatment is total excision when feasible. Otherwise the cyst may be treated with subtotal excision and/or internal anastomosis. When treated properly, these lesions usually have favorable outcomes.
